# Endovascular treatment for ischemic stroke with the drip-and-ship model—Insights from the German Stroke Registry

**DOI:** 10.3389/fneur.2022.973095

**Published:** 2022-08-23

**Authors:** Jan Hendrik Schaefer, Natalia Kurka, Fee Keil, Marlies Wagner, Helmuth Steinmetz, Waltraud Pfeilschifter, Ferdinand O. Bohmann

**Affiliations:** ^1^Department of Neurology, University Hospital Frankfurt, Goethe-University, Frankfurt am Main, Germany; ^2^Department of Neuroradiology, University Hospital Frankfurt, Goethe-University, Frankfurt am Main, Germany; ^3^Department of Neurology, Klinikum Lüneburg, Lüneburg, Germany

**Keywords:** ischemic stroke, endovascular treatment, mechanical thrombectomy, drip-and-ship, direct-to-center

## Abstract

**Background:**

Endovascular therapy (EVT) in acute ischemic stroke has been widely established. Globally, stroke patients are transferred either directly to a thrombectomy center (DC) or a peripheral stroke unit with a “drip-and-ship” (DS) model. We aimed to determine differences between the DS and DC paradigms after EVT of acute stroke patients with large-vessel-occlusion (LVO) in the database of the German Stroke Registry (GSR).

**Methods:**

We performed a retrospective analysis of GSR patients between June 2015 and December 2019 in 23 German centers. Primary outcome was an ordinal shift analysis of modified Rankin Scale (mRS) 90 days after index event. Secondary endpoints included time from symptom onset to recanalization and complications. Tertiary endpoint was the association of imaging strategies in DS admissions with outcome.

**Results:**

2,813 patients were included in the DS and 3,819 in the DC group. After propensity score matching mRS after 90 days was higher in DS than DC admissions (OR 1.26; 95%-CI 1.13–1.40). Time from symptom-onset to flow-restoration was shorter in DC than DS (median 199.0 vs. 298.0 min; *p* < 0.001). DS patients undergoing magnetic resonance imaging (MRI; n=183) before EVT had a lower 90-day mRS than without (*n* = 944) (OR 0.63; 95%-CI 0.45–0.88). ASPECTS assessed on MRI correlated with 90-day mRS (ρ = −0.326; *p* < 0.001).

**Conclusions:**

Clinical outcome was worse for EVT-eligible patients in the DS setting, even though patients were in a better state of health prior to stroke. A potentially mutable factor was the time delay of 99 min from symptom-onset to successful recanalization. Performing MRI before thrombectomy was associated with good outcome and MRI-ASPECTS was negatively correlated with mRS after 90 days.

## Introduction

“Time is brain” has guided stroke care since its inception and equally applies to thrombolysis and endovascular treatment (EVT)(1, 2). Since EVT is oftentimes limited to comprehensive stroke centers the question has emerged whether patients suspected to suffer from acute ischemic strokes should primarily be transferred to hospitals with EVT capabilities (“direct-to-center”, DC) or the nearest peripheral hospital for rapid thrombolysis and in case of large vessel occlusions (LVO) to a thrombectomy center thereafter (“drip-and-ship”, DS). Results from the DAWN and DEFUSE 3 trials provided a framework for EVT in a time window of up to 24 h from symptom onset, rendering secondary transfer feasible in many cases (3, 4). However, the ensuing time delay might negatively influence the eventual outcome. On the contrary, long transportation times to EVT centers are likely to postpone the start of intravenous thrombolysis. Several studies have compared these concepts, some of which have suggested that bypassing stroke units, which only offer intravenous thrombolysis, might confer the benefit of more rapid revascularization and improve outcomes (5). In 2020, a systematic review and meta-analysis of 18 pertinent studies with 7,017 patients demonstrated more functional independence after DC treatment compared to DS (6). Additionally, to save time, patients transferred for EVT from a peripheral center are predominantly directed to angiography without repeat imaging. Recent studies have suggested that foregoing secondary imaging is associated with shortened treatment times and better clinical outcome, but there might be a rationale for additional diagnostic work-up after transport delays (7, 8).

We aimed to analyze the influence of secondary transfers of acute stroke patients and investigate strategies to improve the DS model. To this end, large-scale registries of real-wold data can provide essential insights, and statistical computations such as multivariable regression analysis and propensity score matching can reduce confounding.

## Methods

We retrospectively analyzed data from the German Stroke Registry (GSR, https://www.clinicaltrials.gov identifier NCT0335639), which prospectively collects data on treatment practices, safety and outcome after EVT for ischemic strokes (9, 10). Patients ≥18 years old with acute ischemic stroke in anterior and posterior circulation are enrolled in all participating centers and followed-up for 90 days. No exclusion criteria were defined. Consent is obtained either by the patient or a legal representative. If no consent can be obtained before death, inclusion was based on presumed consent to reduce selection bias. Baseline and treatment data are recorded as part of routine care. Clinical outcome is assessed *via* telephone interview 90 days after stroke. The study protocol was centrally approved by the local Ethics Committee of the Ludwig-Maximilian University Munich (689-15).

Primary endpoint was an ordinal shift analysis of 90-day modified Rankin Scale (mRS) after propensity score matching with multivariable regression analyses (adjusted for age, sex, NIHSS at admission, comorbidities and thrombolysis) (11). Secondary endpoints were a dichotomous analysis of favorable outcome, time delay between DC and DS admissions, correlation between the absolute reduction in National Institute of Health Stroke Scale (NIHSS) from admission to discharge and time from symptom onset and complications in both groups. Tertiary endpoint was the influence of additional imaging [computed tomography (CT), magnetic resonance imaging (MRI)] on treatment times and outcome.

Data analysis was performed using the Statistical Package for Social Sciences (SPSS, version 27.0.1.0, Armonk, N.Y., USA) and R (R package version 3.363) for propensity score matching. Categorical data were evaluated for differences by χ^2^-tests, ordinal and metric data without normal distribution were assessed by Mann-Whitney-*U*-test. According to recommendations of the European Stroke Organization outcome was primarily measured by ordinal logistic regression of mRS as common odds ratios (OR) with adjustment for age, sex, pre-stroke mRS, admission NIHSS, comorbidities and thrombolysis (11, 12). Propensity score matching was performed with 1:1 matching based on the nearest-neighbor algorithm with a caliper width of 0.2 of the propensity score for age, pre-stroke mRS, NIHSS on admission and thrombolysis. The significance level was set to *P* < 0.05, and all tests of hypotheses were two-sided.

## Results

### Study population

Between June 2015 and December 2019, 6,632 patients from 23 centers were enrolled. Of them, 3,819 were treated in the primary admitting center (DC) and 2,813 were transferred after initial treatment in a peripheral hospital (DS). Clinical parameters at baseline for both groups are demonstrated in Table 1. DS patients were more likely to be living unassisted at home (*p* < 0.001) and had lower mRS scores before stroke (median 0 vs. 0; *p* < 0.001). Distribution of comorbidities (arterial hypertension, diabetes, atrial fibrillation, dyslipidemia) did not differ significantly between groups. LVO was more proximal in the DS compared to DC group (extracranial internal carotid artery 7.0 vs. 5.4%; M1-segment 34.1 vs. 32.2%; *p* < 0.001). Clinical outcome after 90 days was available for 2,266 DS and 3,172 DC patients (80.6 vs. 83.1%, *p* = 0.010).

**Table 1:** Baseline, treatment and outcome characteristics for direct-to-center and drip-and-ship admission status, analyzed by χ^2^-test^a^ for categorical data and Mann-Whitney-*U*-test^b^ for continuous, non-Gaussian data.

	**Direct-to-center**	**Drip-and-ship**	***p*-value**
*n*	3,819	2,813	
Age (years, mean ± SD, minimum, maximum)	73.2 ± 13.2 (20–100)	73.0 ± 12.9 (17–100)	0.411^b^
Sex, female, *n* (%)	1,947 (51.0%)	1,414 (50.3%)	0.568^a^
mRS before stroke (median, IQR)	0 (0–1)	0 (0–1)	<0.001^b^
Living status before stroke			
Home	3,074 (80.5%)	2,364 (84.0%)	<0.001^a^
Nursing at home	186 (4.9%)	83 (3.0%)	
Nursing home	277 (7.3%)	170 (6.0%)	
Unknown	282 (7.4%)	196 (7.0%)	
Risk factors			
Arterial hypertension	2,779 (72.8%)	2,063 (73.3%)	0.689^a^
Diabetes	770 (20.2%)	609 (21.6%)	0.241^a^
Atrial fibrillation	1,490 (39.0%)	1,120 (39.8%)	0.876^a^
Dyslipidemia	1,423 (37.3%)	1,044 (37.1%)	0.564^a^
Antithrombotic medication			
Antiplatelets	1,124 (31.8%)	802 (30.4%)	0.251^a^
VKA	263 (7.4%)	218 (8.3%)	0.230^a^
DOAC	480 (13.6%)	350 (13.3%)	0.459^a^
NIHSS on admission (median, IQR)	14.0 (9–18)	15.0 (10–19)	0.003^b^
Vessel occlusion			
ICA extracranial	207 (5.4%)	198 (7.0%)	<0.001^a^
ICA intracranial	753 (19.8%)	617 (20.8%)	
MCA M1	1,228 (32.2%)	960 (34.1%)	
MCA M2	786 (20.6%)	552 (19.6%)	
ACA	86 (2.3%)	63 (2.2%)	
PCA	121 (3.2%)	47 (1.7%)	
BA	385 (10.1%)	276 (9.8%)	
VA	79 (2.1%)	45 (1.6%)	
Stroke etiology			
Cardioembolic	1,822 (47.7%)	1,379 (49.0%)	<0.001^a^
Large-vessel-disease	867 (22.7%)	639 (22.7%)	
ESUS	588 (15.4%)	486 (17.3%)	
Dissection	68 (1.8%)	45 (1.6%)	
Other	180 (4.7%)	98 (3.5%)	
Unknown	294 (7.7%)	166 (5.9%)	
Thrombolysis	1,767 (46.3%)	1,556 (55.3%)	<0.001^a^

Mean and standard deviation (SD) or median and interquartile range (IQR) are shown as appropriate. mRS, modified Rankin Scale; NIHSS, National Institute of Health Stroke Scale; VKA, vitamin K antagonist; DOAC, direct oral anticoagulant; ICA, internal carotid artery; MCA, middle cerebral artery; ACA, anterior cerebral artery; PCA, posterior cerebral artery; BA, basilar artery; VA, vertebral artery; ESUS, embolic stroke of unknown source.

### Primary endpoint

**Figure 1:**
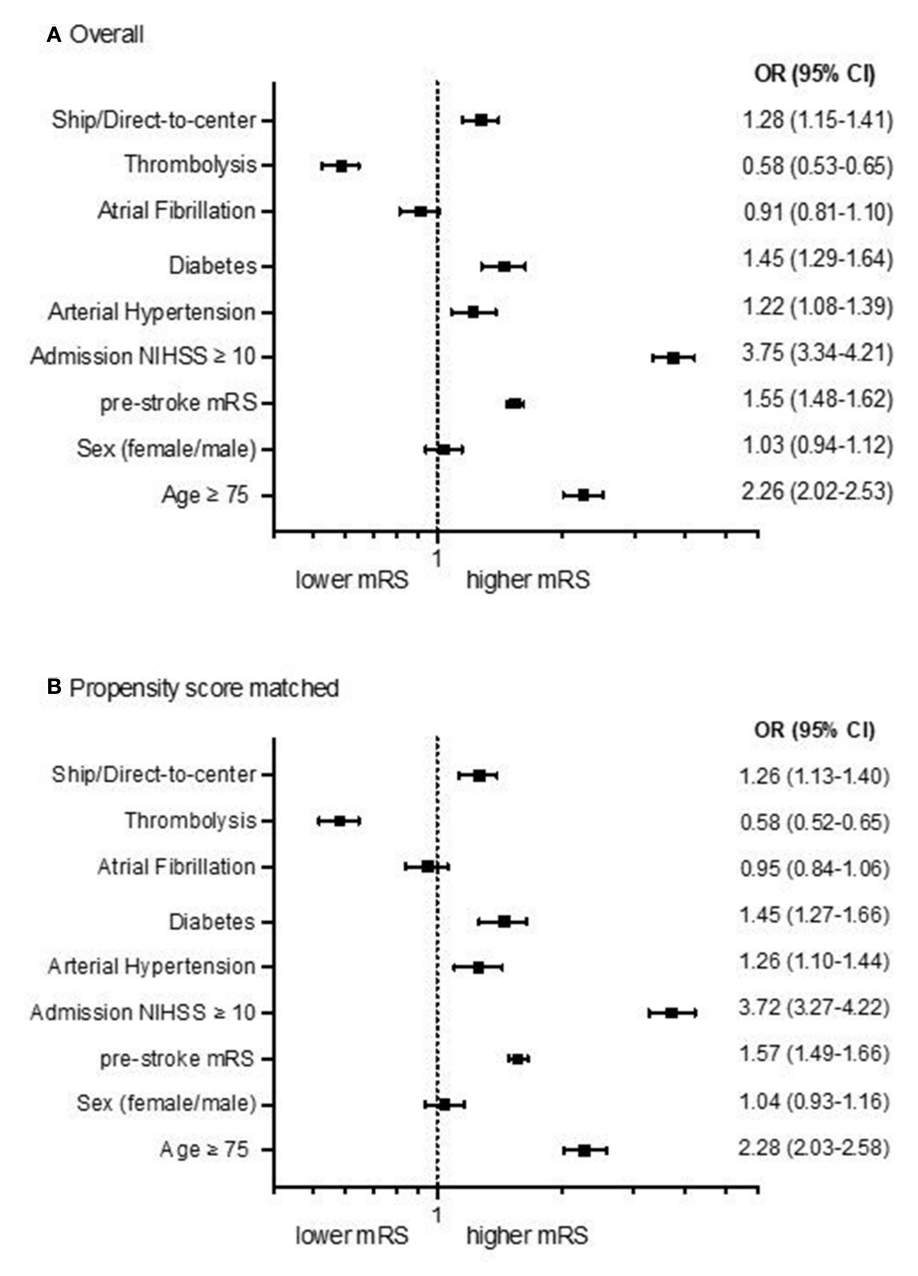
Forest plots showing multivariable ordinal regression analyses of the adjusted common odds ratio (OR) of modified Ranking Scales (mRS) 90 days after stroke for DC/DS admission status, sex, age, admission NIHSS, comorbidities and thrombolysis in all patients [**(A)**, *n* = 5,107] and after propensity score matching [**(B)**, *n* = 4,348]. The OR is presented logarithmically with 95%-confidence intervals.

**Figure 2:**
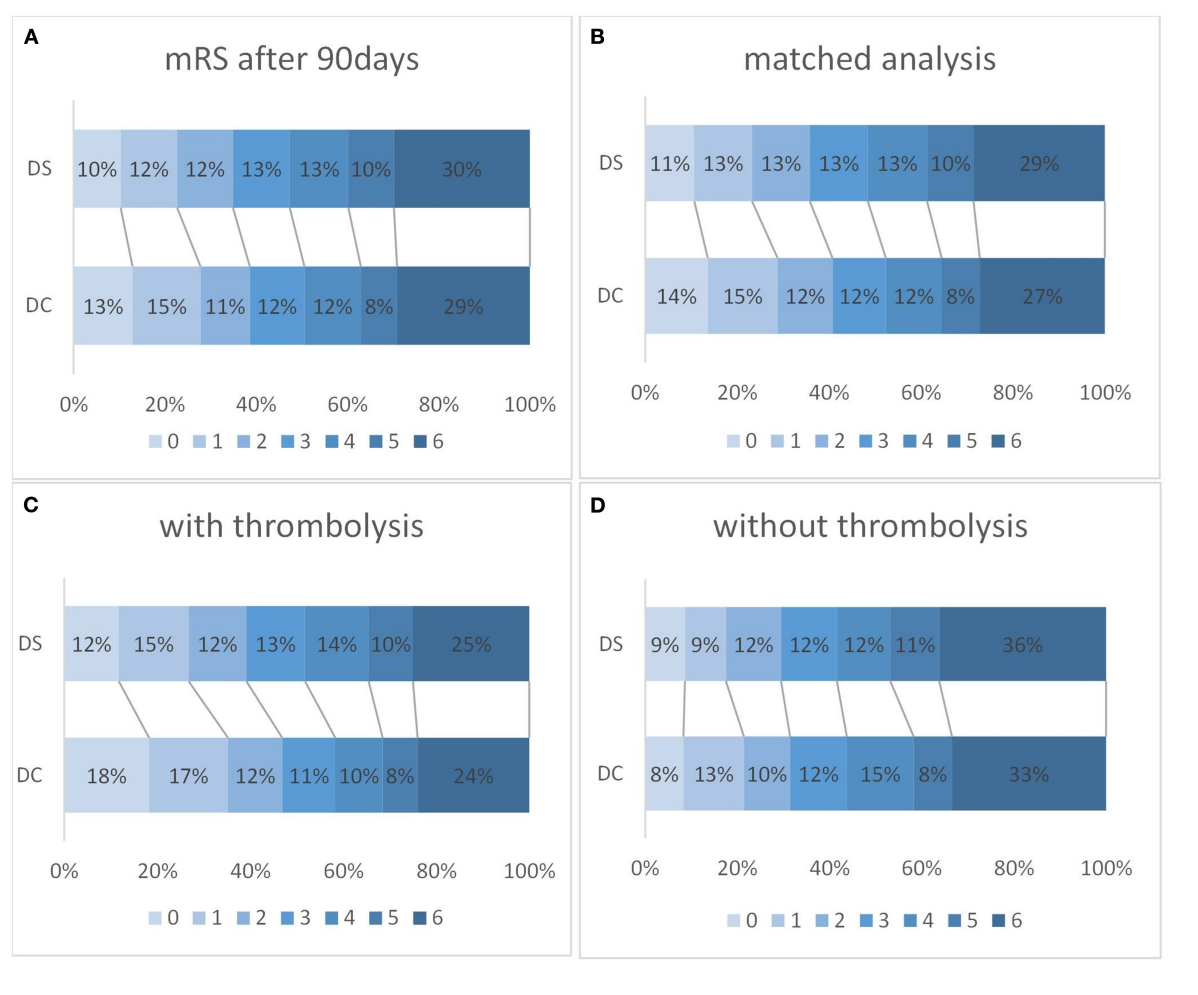
The outcome on modified Rankin Scale (mRS) after 90 days for drip-and-ship (DS) and direct-to-center (DC) in all patients [**(A)**; DS *n* = 2,266; DC, *n* = 3,172; OR 1.28; 95%-CI 1.15–1.41; *p* < 0.001] and after a propensity score matched analysis [**(B)**; DS *n* = 2,114; DC *n* = 2,993; OR 1.28; 95%-CI 1.15–1.41; *p* < 0.001]. Favorable outcome (mRS 0–2) was also significantly more likely in DC than DS admissions after thrombolysis [**(C)**; DS *n* = 1,273, DC *n* = 1,485; OR 0.73; 95%-CI 0.64–0.83; *p* < 0.001], but not if thrombolysis was not administered [**(D)**; DS *n* = 977, DC *n* = 1,663; OR 0.87; 95%-CI 0.72–1.05; *p* = 0.149].

After multivariable regression analysis mRS after 90 days was higher in DS than DC admissions [odds ratio (OR) 1.26; 95%-confidence interval 1.13–1.40; *p* < 0.001; Figure 1A]. Propensity score matching balanced the baseline characteristics of both groups (Table 2), but OR for higher mRS scores after 90 days was still significantly lower in DC compared to DS admissions (DC *n* = 2,234; DS *n* = 2,114; OR 1.26; 95%-CI 1.13 to 1.40; *p* < 0.001; Figures 1B, 2B).

**Table 2:** Results from baseline characteristics, treatment procedures and outcome of direct-to-center and drip-and-ship patients after propensity score matching for age, pre-stroke mRS, NIHSS at admission and thrombolysis.

**Propensity score matched analysis**			
	**Direct-to-center**	**Drip-and-ship**	***p***
*N*	2,556	2,555	
Mean age (years ± SD, minimum, maximum)	73.1 ± 13.1 (21–100)	72.8 ± 12.9 (17–99)	0.417^b^
Sex, female (*n*, %)	1,293 (50.6%)	1,282 (50.2%)	0.780^a^
mRS before stroke (median, IQR)	0 (0–1)	0 (0–1)	0.557^b^
Risk factors			
Arterial hypertension	1,969 (77.3%)	1,959 (77.2%)	0.920^a^
Diabetes	527 (20.7%)	569 (22.4%)	0.152^a^
Atrial fibrillation	1,044 (41.1%)	1,056 (41.5%)	0.754^a^
Dyslipidemia	1,006 (39.6%)	992 (39.1%)	0.730^a^
NIHSS on admission (median, IQR)	15.0 (10–19)	15.0 (10–19)	0.910^b^
Imaging (*n*, %)			
CT	2,373 (93.0%)	2,291 (92.0%)	0.198^a^
MRI	239 (9.4%)	208 (8.4%)	0.216^a^
Stroke etiology			
Cardioembolic	1,301 (51.4%)	1,316 (51.9%)	0.373^a^
Large-vessel-disease	626 (24.7%)	618 (24.4%)	
ESUS	56 (2.2%)	45 (1.8%)	
Dissection	429 (16.9%)	463 (18.2%)	
Other	117 (4.6%)	93 (3.7%)	
Thrombolysis	1,407 (55.0%)	1,430 (55.9%)	0.518^a^
Symptom onset—admission in center (minutes, median, IQR)	63.0 (45.0–103.0)	195.0 (149.0–255.0)	<0.001^b^
Symptom onset—thrombolysis (minutes, median, IQR)	90 (70.0–120.0)	95 (71.0–180.0)	0.083^b^
Symptom onset—flow restoration (minutes, median, IQR)	199.0 (160.0–258.0)	298.0 (239.8–370.0)	<0.001^b^
NIHSS after 24 h (median, IQR)	10 (4–19)	11 (5–19)	0.001^b^
ICH after 24 h	267 (10.4%)	329 (12.9%)	0.007^b^
ICH between 24 h and discharge	77 (3.0%)	99 (3.9%)	0.107^b^
NIHSS on discharge (median, IQR)	5 (1–13)	6 (2–14)	<0.001^b^
mRS on discharge (median, IQR)	4 (2–5)	4 (2–5)	<0.001^b^
mRS after 90 days (median, IQR)	3 (1–6)	4 (2–6)	0.001^b^

Analysis was performed by χ^2^-test^a^ for categorical data and Mann-Whitney-U-test^b^ for continuous, non-Gaussian data. Mean and standard deviation (SD) or median and interquartile range (IQR) are shown as appropriate.

### Secondary endpoints

A favorable clinical outcome after 90 days, defined as mRS 0–2, was less frequent in the DS compared to the DC group (34.9 vs. 38.7%; OR 0.73; 95%-CI 0.64–0.83; *p* < 0.001). After intravenous thrombolysis favorable outcomes were more frequently observed, however still less so in the DS than the DC group (39.1 vs. 46.9%; *p* < 0.001; Figures 2A–D). Without thrombolysis, no significant difference in favorable outcome could be detected between DS and DC (29.5 vs. 31.4%; OR 0.87; 95% CI 0.72–1.05; *p* = 0.149).

**Figure 3:**
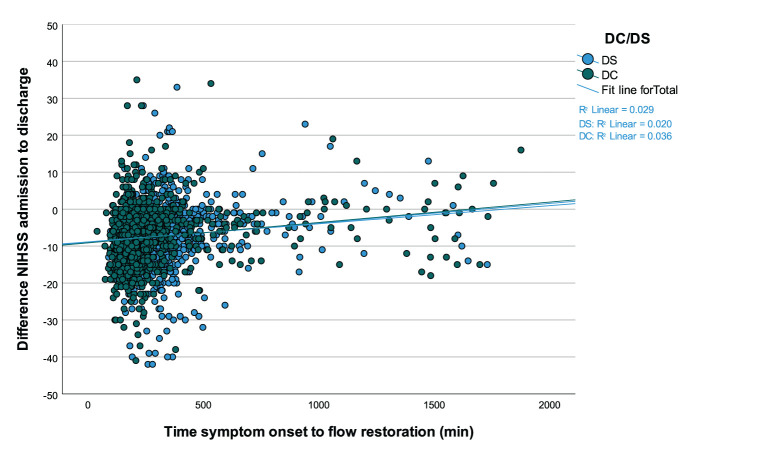
Correlation between the time from symptom onset to successful endovascular recanalization and the absolute difference of NIHSS scores at hospital admission and discharge. Values <0 signify a benefit from thrombectomy and values ≥0 suggest lack of benefit, which becomes more likely along with passing time in minutes for drip-and-ship (DS) and direct-to-center treated patients (*n* = 2,366; ρ = 0.27; *p* < 0.001).

After propensity score matching, median time interval between symptom onset and flow restoration was 199.0 min (IQR 160.0–258.0 min) for DC and 298.0 min (IQR 239.8–370.0 min) for DS admissions (*p* < 0.001). Overall, there was a weak albeit significant correlation between the time from stroke onset to successful endovascular recanalization and the net clinical benefit as measured by the difference of NIHSS from admission to discharge (*n* = 2,366; Spearman's ρ = 0.27; *p* < 0.001) (Figure 3).

Complications were more common in the DS than the DC group (any adverse event: 39.6 vs. 34.1%; *p* < 0.001) (Table 3). Notably, intracranial hemorrhages were recorded more frequently in the DS group during EVT (3.2 vs. 2.4%; *p* = 0.039) and 24 h after intervention (12.6 vs. 10.0%; *p* = 0.001). Contrarily, early recurrent strokes were less frequent in the DS than the DC group (2.1 vs. 5.3% vs.; *p* < 0.001).

**Table 3:** Results from baseline characteristics, treatment procedures and outcome of direct-to-center and drip-and-ship patients after propensity score matching.

	**Direct-to-center**	**Drip-and-ship**	***p*-value**
Final TICI			
0	308 (8.1%)	223 (7.9%)	0.035^b^
1	51 (1.3%)	44 (1.6%)	
2a	205 (5.4%)	156 (5.5%)	
2b	1,189 (31.1%)	978 (34.8%)	
3	1,954 (51.2%)	1,369 (48.7%)	
ICH during MT	90 (2.4%)	90 (3.2%)	0.039^a^
ICH after 24 h	381 (10.0%)	354 (12.6%)	0.001^a^
ICH between 24 h and discharge	118 (3.1%)	106 (3.8%)	0.132^a^
Recurrent stroke after 24 h	203 (5.3%)	59 (2.1%)	<0.001^a^
Recurrent stroke between 24 h and discharge	62 (1.6%)	37 (1.3%)	0.357^a^
Malignant media infarction after 24 h	118 (3.1%)	108 (3.8%)	0.100^a^
Malignant media infarction between 24 h and discharge	98 (2.6%)	91 (3.2%)	0.117^a^
Median NIHSS after 24 h (IQR)	10 (4–19)	12 (5–19)	<0.001^b^
NIHSS on discharge (median, IQR)	5 (2–14)	6 (1–13)	<0.001^b^
NIHSS admission-discharge (median, IQR)	−6 (−11 to −1)	−5 (−10 to 0)	0.003^b^
mRS on discharge (median, IQR)	3 (2–5)	4 (2–5)	<0.001^a^
mRS after 90 days (median, IQR)	3 (1–5)	3 (1–5)	0.003^a^
Symptom onset—admission in center (minutes, median, IQR)	65 (49.0–107.0) (*n* = 2,052)	195.0 (149.0–254.0) (*n* = 1,738)	<0.001^b^
Admission—groin puncture (minutes, median, IQR)	82.0 (61.0–159.0) (*n* = 3,415)	48.0 (31.0–114.0) (*n* = 2,624)	<0.001^b^
Groin puncture—flow restoration (minutes, median, IQR)	41.0 (26.0–101.0) (*n* = 3,137)	41.0 (26.0–98.0) (*n* = 2,429)	0.758^b^
Symptom onset—flow restoration (minutes, median, IQR)	201.0 (160.0–380.0) (*n* = 1,740)	297.5 (240.0–477.0) (*n* = 15,05)	<0.001^b^
Symptom onset—thrombolysis (minutes, median, IQR)	90.0 (72.0–175.0) (*n* = 1,186)	95.0 (71.0–180.0) (*n* = 897)	0.220

Analysis was performed by χ^2^-test^a^ for categorical data and Mann-Whitney-U-test^b^ for continuous, non-Gaussian data. Mean and standard deviation (SD) or median and interquartile range (IQR) are shown as appropriate. ICH, intracranial hemorrhage.

### Tertiary endpoint

**Figure 4:**
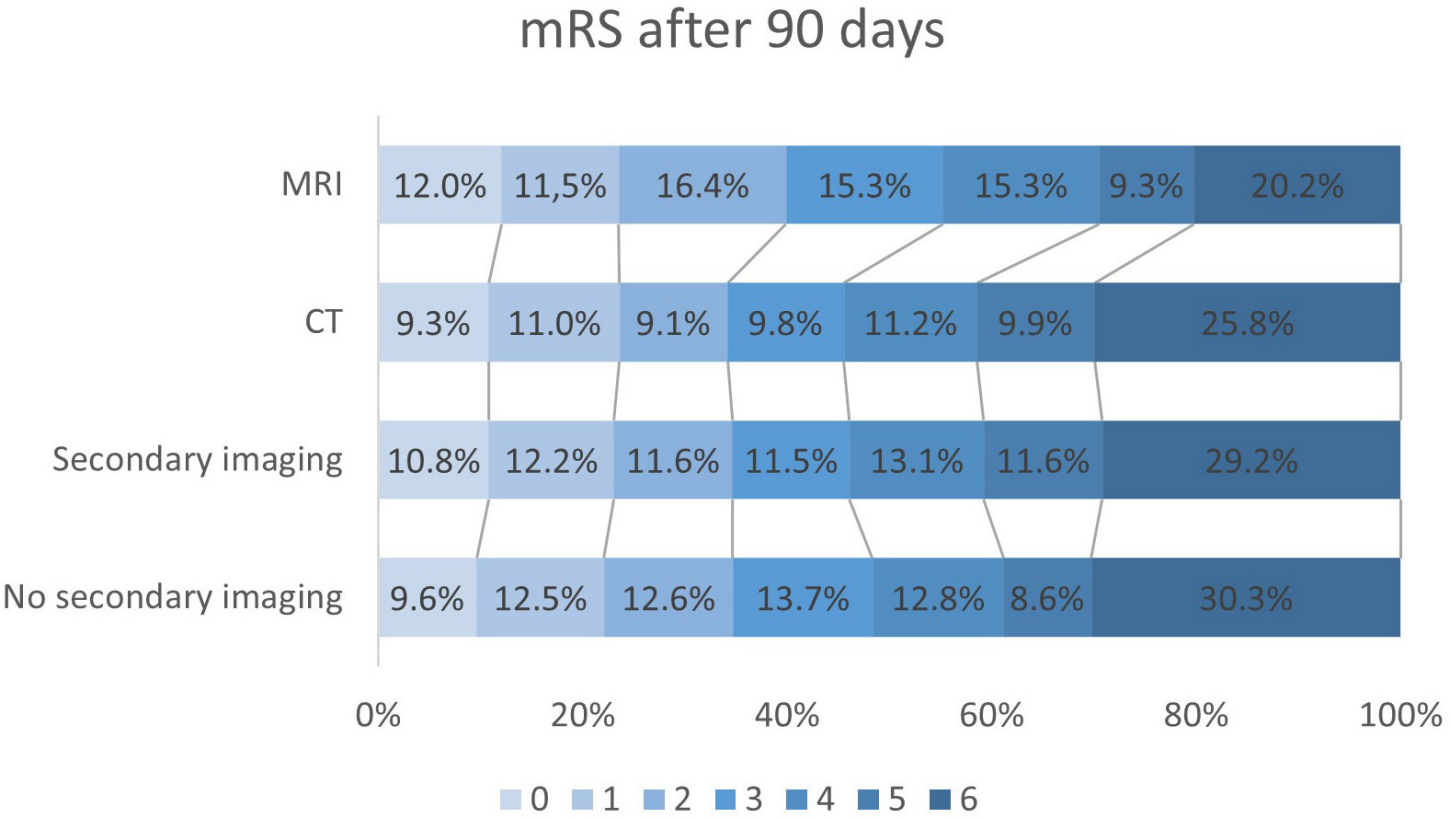
Modified Ranking scale (mRS) outcome data after 90 days in all drip-and-ship (DS) patients without secondary imaging (*n* = 1,171), with secondary imaging in interventional center (*n* = 1,045), only CT-imaging (*n* = 944) and MRI (*n* = 183). Secondary imaging in general did not significantly affect mRS compared to only initial imaging in peripheral centers (*p* = 0.206). After multivariable adjustment DS patients undergoing MRI before EVT had lower mRS scores than those without (OR 0.63; 95%-CI 0.46–0.88; *p* = 0.006).

DS patients with MRI were more likely to achieve a lower mRS score after 90 days, as evidenced by multivariable regression analysis with adjustment for pre-stroke mRS, gender, Alberta Stroke Program Early CT Score (ASPECTS) and thrombolysis (*n* = 183 vs. *n* = 944 without MRI; OR 0.634; 95%-CI 0.46–0.88; *p* = 0.006) (Figure 4). CT-ASPECTS correlated weaker with the 90-day mRS (*n* = 1,480, Spearman's ρ = −0.139; *p* < 0.001) than MRI-ASPECTS (*n* = 108, Spearman's ρ = −0.326; *p* < 0.001). Any secondary imaging led to a median delay of 28 min between admission and groin puncture, and MRI increased this interval compared to CT from 60 min to 82 min (*p* < 0.001; Table 4).

**Table 4:** Baseline characteristics and procedure times of drip-and-ship admissions according to whether secondary imaging was performed and if there was CT only or MRI available.

	**No secondary imaging**	**Secondary imaging**	***p***	**CT only**	**MRI**	***p***
*n*	1,171	1,045		944	183	
Mean age (years ± SD)	73.9 ± 12.5	72.2 ± 13.0	0.020^b^	72.7 ± 12.7	70.6 ± 13.6	0.060^b^
Sex (female; *n*; %)	606 (51.8%)	510 (48.9%)	0.173^b^	468 (49.6%)	86 (47.0%)	0.519^b^
Median mRS before stroke (IQR)	0 (0–1)	0 (0–1)	0.032^a^	0 (0–1)	0 (0–1)	0.580^b^
NIHSS on admission (median, IQR)	15.0 (10.0–19.0)	15.0 (9.0–19.0)	0.007^b^	15.0 (9.0–19.0)	12.0 (7.0–17.0)	0.004^b^
ASPECTS (median, IQR)	9.0 (8.0–10.0)	9.0 (7.0–8.0)	<0.001^b^	8.0 (7.0–9.0)	7.5 (6.0–9.0)	0.027^b^
Thrombolysis (*n*; %)	653 (56.4%)	588 (56.3%)	0.996^a^	544 (57.6%)	90 (49.5%)	0.050^a^
Symptom onset—admission in center (minutes, median, IQR)	187.0 (145.0–242.8)	198.0 (148.0–255.0)	0.035^b^	194.0 (147.0–250.5)	240.0 (190.0–316.0)	<0.001^b^
Admission—groin puncture (minutes, median, IQR)	34.0 (25.0–48.0)	62.0 (46.0–86.0)	<0.001^b^	60.0 (44.0–81.0)	82.0 (50.0–117.0)	<0.001^b^
Admission—flow restoration (minutes, median, IQR)	77.0 (57.0–109.0)	113.0 (87.0–148.0)	<0.001^b^	111.0 (85.0–143.0)	130.0 (96.0–180.0)	<0.001^b^
Groin puncture—flow restoration (minutes, median, IQR)	38.0 (25.0–61.0)	43.0 (28.0–70.0)	0.001^b^	43.0 (27.0–70.0)	40.0 (27.0–69.0)	0.761^b^
Symptom onset—flow restoration (minutes, median, IQR)	270.0 (219.0–345.0)	313.0 (260.0–386.3)	<0.001^b^	310.0 (255.0–375.0)	381.0 (334.8–517.8)	<0.001^b^

Analysis was performed by χ^2^-test^a^ for categorical data and Mann-Whitney-U-test^b^ for continuous, non-Gaussian data. Mean and standard deviation (SD) or median and interquartile range (IQR) are shown as appropriate.

## Discussion

In this retrospective analysis DS patients fared worse in terms of functional neurological outcome 90 days after stroke compared to DC admission. This difference persisted after propensity score matching and adjustment for confounding factors. The most likely causative factor was a median time delay from symptom onset to reperfusion of 99 min between DC and DS. The risk of intracranial hemorrhages after recanalization was also higher in DS than DC (19.6 vs. 15.4%), a finding which might be attributed to more reperfusion injury after delayed recanalization. Other stroke registries have already stressed the time advantage of the DC strategy with a median delay of 109 and 96 min from symptom onset to recanalization, respectively (5, 13). A median time delay of 99 min as in our study would correspond to the loss of roughly 188 million neurons per patient according to a modeled analysis (14).

Considering these disadvantages of DS, should ambulances be instructed that “routing is brain” or is there potential for improvement of the paradigm? Based on clinical data, statistical models have been developed to determine which proximity of EVT centers and procedure times would justify bypassing thrombolysis-only hospitals (15). A probabilistic sensitivity analyses suggest that bypassing peripheral stroke units may achieve better functional outcomes, unless it delays thrombolysis by more than 30 min in urban and 50 min in rural areas (16).

This strategy has been put to test in the recently published RACECAT trial (17). Here, neurological disability of patients, who were preclinically randomized to either be transported directly to a thrombectomy center or the closest stroke unit based on a clinical score indicating LVO, was not different after 90 days. However, mRS > 2 and admission > 7 h from symptom onset were exclusion criteria, which differs from real-world data and would exclude about 40% of patients in our study (18).

In general, time delay is a modifiable by logistic optimization. Pre-hospital delays have an influence in both settings, as evidenced by a GSR analysis with focus on treatment times, which showed an increase in unfavorable outcome with every additional hour from onset to admission for DC- and DS-treated patients (absolute risk difference +0.7 vs. +1.3%) (19). An analysis of a federal stroke registry in Germany demonstrated onset-to-EVT times in DS patients were considerably shortened by approximately 100 min since 2012, most likely allowing for better clinical outcomes in the future (20).

However, even with shortened transport-to-center times, many patients are still likely to arrive in a late time window, i.e., >6 h after symptom onset or >2 h after initial imaging, prompting the question whether to repeat imaging. Our results suggest that DS patients undergoing MRI before EVT were more likely to achieve a good outcome despite longer intervals between symptom onset and recanalization. This appears likely to represent a selection bias by excluding patients who subsequently did not receive EVT due to presumed lack of salvageable tissue. The approximate intrahospital time delay caused by MRI was 22 min compared to CT and 48 min to no secondary imaging. Hence, it is important to consider whether the associated loss of salvageable tissue can be legitimized by cases, in which patients can be spared an unnecessary thrombectomy with inherent intra- and post-procedural risks (21). Such futile interventions could possibly be reduced by MRI and ASPECTS can guide prognostication, which might then outweigh the disadvantage of time consumption (22). Considering the recently published RESCUE-JAPAN results, even patients with a MRI-ASPECTS of 3–5 can benefit from EVT compared to medical treatment alone, but it is important to note that in a time window of >6 h after symptom onset MRI mismatch between diffusion-weighted-imaging and fluid attenuation inversion recovery was utilized (23). Besides MRI, mismatch imaging with CT-perfusion might also provide clinical information as a basis for decision-making. A comprehensive registry or prospective studies of secondary imaging after transfer regardless of whether subsequent EVT was performed would certainly provide better evidence for clinical decision making.

There are several limitations to this study. Firstly, only EVT was included, therefore no definite conclusion on transfer strategies for all stroke patients can be formed. Due to the voluntary nature of participation in the GSR a possible selection bias cannot be excluded. However, compared to other registries which are limited to preformed stroke networks, the GSR resembles real-world conditions more closely due to decentralization. No distances between the patient's location and admitting stroke center were recorded, but the time between onset and admission is likely to be a more relevant factor considering different transport mechanisms. Another difference in baseline parameters between our DC and DS group were more proximal vessel occlusions in the latter, which could partly explain the less favorable results. Lastly, there was a difference in the availability of follow-up information after 90 days between groups, for which possible factors such as death cannot be ruled out.

## Conclusions

Secondary transfer led to worse outcome compared to direct-to-center EVT. Considering the similar correlation between time to recanalization and NIHSS reduction in both groups, optimizing EVT workflows to reduce time delay remains a crucial factor to improve the DS paradigm. Despite late recanalization, patients with MRI showed favorable outcomes compared to no secondary imaging and MRI-ASPECTS correlated negatively with mRS after 90 days, suggesting that in certain settings (e.g., after prolonged transfer) MRI could augment prognostic accuracy, which in turn could facilitate a better definition of the patient collective most likely to benefit despite delayed EVT and thus reduce futile interventions.

## Data availability statement

The raw data supporting the conclusions of this article can be provided upon reasonable request.

## Ethics statement

The studies involving human participants were reviewed and approved by Ethics Committee of the Ludwig-Maximilian University Munich (689-15). The patients/participants provided their written informed consent to participate in this study.

## Author contributions

JS, WP, and FB researched literature and conceived the study. JS, NK, and FB gathered and analyzed the data. All authors reviewed and edited the manuscript and approved the final version of the manuscript.

## Conflict of interest

Author FB reports receiving research grant from Stryker Neurovascular and Boehringer Ingelheim and speaker's honoraria from Alexion, Medtronic and Laerdal. Author WP reports speaker's honoraria from Laerdal. The remaining authors declare that the research was conducted in the absence of any commercial or financial relationships that could be construed as a potential conflict of interest.

## Publisher's note

All claims expressed in this article are solely those of the authors and do not necessarily represent those of their affiliated organizations, or those of the publisher, the editors and the reviewers. Any product that may be evaluated in this article, or claim that may be made by its manufacturer, is not guaranteed or endorsed by the publisher.
